# A case report of an excellent response to interferon- α in a patient with functional metastasized neuroendocrine tumor refractory to other treatments

**DOI:** 10.1097/MD.0000000000020820

**Published:** 2020-06-19

**Authors:** Burcin Özdirik, Frank Tacke, Fabian Benz, Holger Amthauer, Uli Fehrenbach, Christoph Roderburg, Henning Jann

**Affiliations:** a Charité – University Medicine Berlin, Department of Hepatology and Gastroenterology, Campus Virchow Klinikum and Charité Campus Mitte; b Charité – University Medicine Berlin, Department of Nuclear Medicine, corporate member of Freie Universität Berlin, Humboldt-Universität zu Berlin and Berlin Institute of Health, Klinik für Nuklearmedizin; c Charité – University Medicine Berlin, Department of Radiology, corporate member of Freie Universität Berlin, Humboldt-Universität zu Berlin, and Berlin Institute of Health, Klinik für Radiologie, Berlin, Germany.

**Keywords:** antiproliferative, antisecretory, case report, efficacy, interferon, neuroendocrine tumor, toxicity

## Abstract

**Introduction:**

Interferon alpha (IFNα) has been used for a long time in patients with functionally active neuroendocrine tumors (NET). However, due to the unfavorable toxicity profile of interferon, the perceived limited efficacy as well as the development of novel substances, IFNα is only used sparingly in the treatment of NET to date.

**Patients concerns and diagnosis:**

We describe the case of a 63-year-old male patient with highly differentiated, functional NET of the ileum and synchronous liver metastasis.

**Interventions:**

After failure of classical therapies including dose-intensified somatostatin analog treatment and palliative primary tumor resection, a therapy with pegylated IFNα2a (135 μg/wk) was initiated. Following this treatment, the patient fully recovered from signs of hypersecretion and demonstrated an impressive tumor response.

**Outcomes:**

Thirty months after initiating IFNα, the patient is still free of clinical symptoms and shows a sustained tumor response. Notably, no relevant side effects were observed.

**Conclusion:**

Our case report supports the use of IFNα in patients with functional NET refractory to classical treatments.

## 1 Introduction

Neuroendocrine neoplasia (NEN) comprise tumors that are derived from the diffuse endocrine system. NEN are very heterogeneous in terms of clinical symptoms and patients’ prognosis. While patients with well differentiated tumors display an extraordinary favorable prognosis, patients with undifferentiated tumors face a prognosis that is comparable or even worse to that of small cell lung carcinoma. In patients with non-functional diseases, NEN are mostly asymptomatic, while patients with hormonal hypersecretion might display a so called carcinoid syndrome, which includes diarrhea, abdominal pain, flushing, bronchospasm and carcinoid heart disease.^[1–3]^

Surgical resection is the only curative treatment for neuroendocrine tumors (NET), although in over 50% of patients, NET are metastasized at the time of diagnosis.^[4]^ In non-resectable cases, according to current guidelines, somatostatin analog (SSAs) represent the first-line therapy for patients with functional disease to achieve symptomatic relief and inhibit tumor growth.^[5]^ On a molecular level, the function of SSA can be explained by binding of SSA to somatostatin receptors (SSR) localized on the tumor cell surface. Currently available SSA provide only moderate antiproliferative effects but effectively inhibit hormonal secretion and ameliorate flushing and diarrhea in about 75% of patients with carcinoid syndrome. Although there are no randomized studies comparing different SSA, the efficacy of available substances seems to be similar.^[6]^ In the case of insufficiently controlled carcinoid syndrome, various therapeutic modalities including systemic and locoregional treatments are available which, however, have not been compared with each other, and whose efficacy can only be assessed empirically. In many centers, high-dose SSA therapy is given in patients with insufficient response to standard SSA.^[7,8]^ Despite the toxicity of this concept is low in most cases, prospective trials proving the efficacy high-dose SSA in this indication are lacking. Besides escalating the SSA dose, symptom control can also be achieved by shortening the injection interval for example, 3 weeks.^[9–11]^ Moreover, the orally available serotonin synthesis inhibitor telotristatetiprat (or telotristatethyl) can relieve diarrhea associated with carcinoid syndrome.^[12]^

Interferon alpha (IFNα) has been used for a long time in patients with functionally active NETs. Administration of IFNα is associated with an inhibition of hormone secretion and a clinical improvement in up to 70% of NEN-patients.^[13]^ Despite large phase III- trials analyzing the efficacy of IFNα in NET are lacking, several smaller studies have shown response rates in up to 27% of patients and tumor stabilization rates of 40% upon treatment with IFNα.^[6]^ However, 2 randomized studies failed to demonstrate a superiority of a combination of IFNα and SSA compared to SSA alone.^[14]^ Nevertheless, in comparison to SSA, IFNα was associated with significantly higher rates of adverse effects (loss of appetite, weight loss, fatigue, bone marrow depression, hepatotoxic reaction, depressive syndrome, mental changes, visual disturbances). Thus, based on its perceived limited efficacy and an unfavorable toxicity profile, the use of IFNα in patients with NET has considerably diminished in the last years. We herein report the case of a patient with functional ileal NET grade 2 (NET G2) and synchronous liver metastases, who received IFNα therapy after intensified SSA monotherapy and tumor debulking failed, resulting in a complete recovery from symptoms of hormone hypersecretion and a sustained tumor response of 30 months without any serious adverse events.

## 2 Case presentation

We report the case of a 63-year-old male patient who was diagnosed in July 2016 with a highly differentiated functional NET of the ileum and synchronous liver metastases. An overview of the entire course of disease (including information on staging, key diagnostics, therapies and response) is provided in Table 1.

**Table 1:**
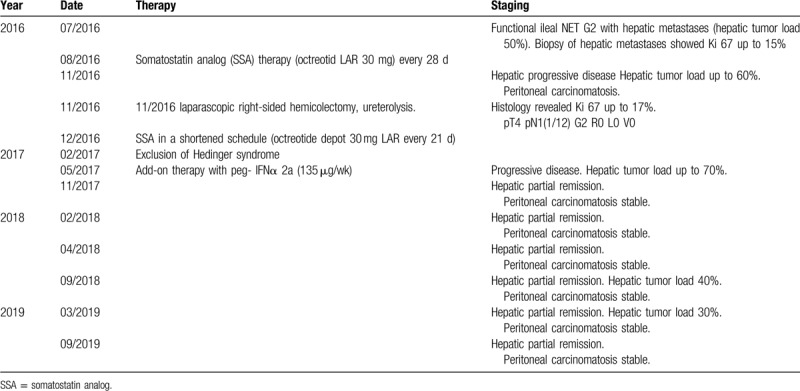
Course of disease.

**Figure 1:**
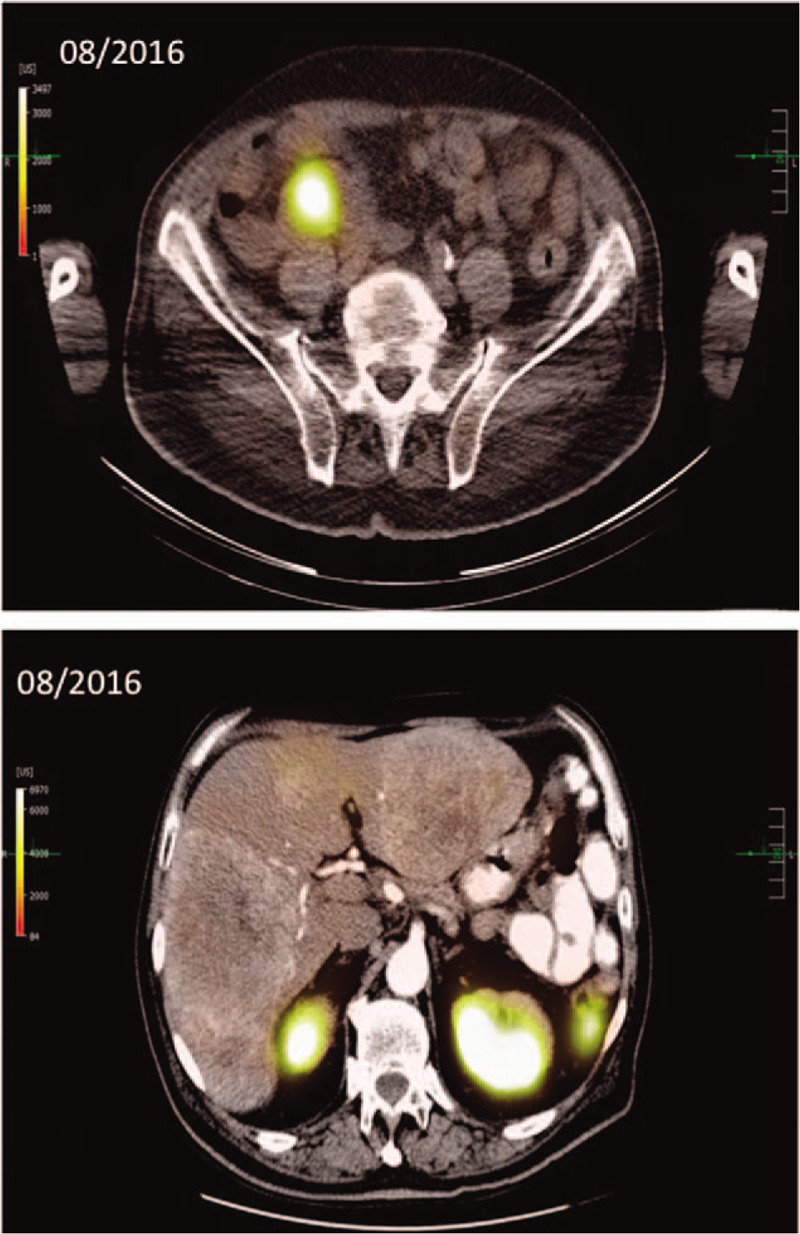
Initial DOTATOC-PET/CT imaging of a functional ileal NET G2 with synchronous hepatic metastases reveals: A) Somatostatin-Receptor (SSR) positive ileal primary tumor and B) several SSR negative hepatic metastases (initial hepatic tumor load 50%). PET/CT = positron emission tomography/computed tomography. SSR = somatostatin receptor.

**Figure 2:**
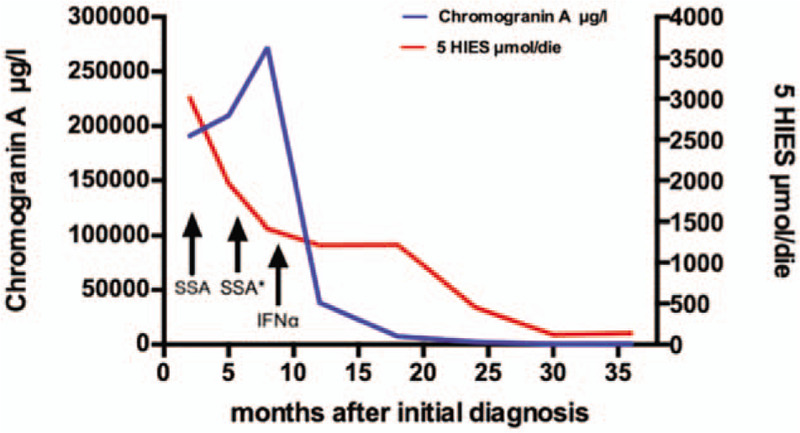
Chromogranin A concentration in serum and urinary 5-hydroxyindoleacetic acid concentration during the course of treatment. The graph illustrates a drastic reduction of chromogranin A in serum and urinary 5-hydroxyindoleacetic acid concentrations after initiation of therapy with IFNα2a while only a slightly decrease was demonstrated during therapy with SSA monotherapy and SSA therapy in a shortened interval (octreotide depot 30 mg LAR every 21 d). LAR = long-acting release, SSA = somatostatin analog.

When presenting at our outpatient unit, the patient was suffering from hot flushes and diarrhea (up to 10x/day) since several years. An ultrasonography performed by the family practitioner of the patient had revealed several echogenic liver lesions. We conducted a multi-slice computed tomography (CT) revealing multiple liver lesions (initial hepatic tumor load 50%) and a tumor formation of the ileum. A subsequently performed DOTATOC- positron emission tomography/computed tomography detected SSR positivity for the primary tumor but multiple SSR negative liver metastases (Fig. 1). In further work-up, serum Chromogranin A concentrations were significantly elevated (29,924 μg/L) and urinary 5-hydroxyindoleacetic acid (5-HIAA) excretion was increased to 10,915 mg/24 h (Fig. 2). Immunochemical analysis of a biopsy taken from a liver metastasis revealed a strong expression of both synaptophysin and chromogranin, ultimately proving the presence of a NET differentiation. The proliferation rate according to antigen KI-67 was 10% to 15%.

**Figure 3:**
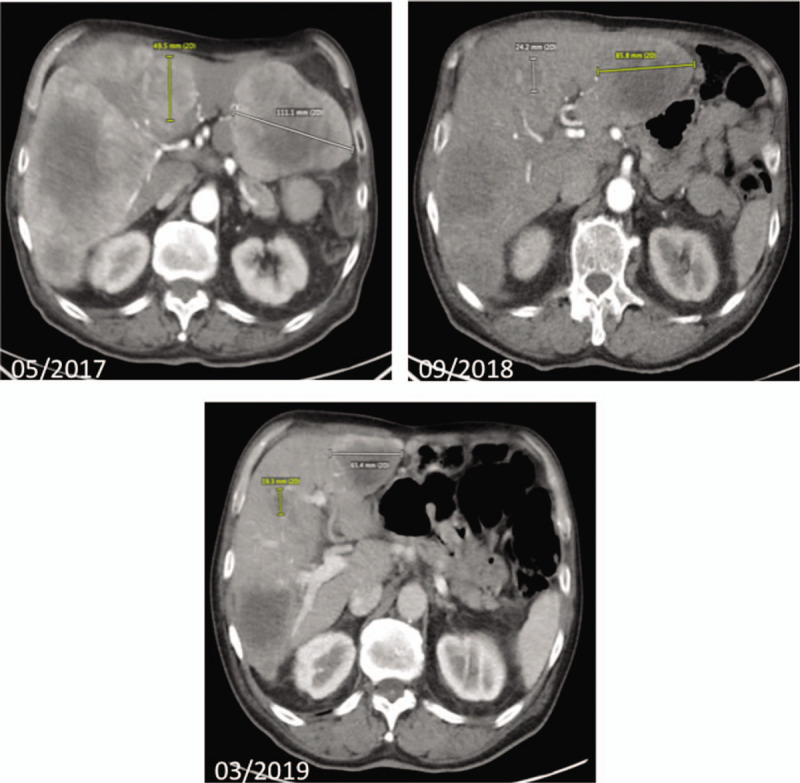
Hepatic metastases in abdominal CT - imaging before (05/2017) and during (09/2018, 03/2019) treatment with IFNα2a. CT imaging before initiation of treatment (05/2017) depicts a hepatic tumor load of 70%, whereas CT imaging in march 2019 shows a significant reduction of hepatic tumor load to 30% (03/2019). CT = computed tomography.

We initiated SSA therapy (octreotide depot 30 mg LAR every 28 days) in August 2016. Follow up CT imaging in November 2016 revealed progressive disease showing a tumor progression occurring in the liver (tumor load up to 60%). To prevent a potentially life-threatening small bowel obstruction, the patient was admitted to laparascopic right-sided hemicolectomy along with right sided ureterolysis. Intraoperatively, peritoneal lesions were detected that turned out as metastases of a neuroendocrine tumor (antigen KI-67 17%), confirming peritoneal carcinomatosis. In this clinical constellation (progression and unresolved flushing under standard dose SSA), we decided to administer SSA therapy in a shortened schedule (octreotide depot 30 mg LAR every 21 days) trying to increase its efficacy. Nevertheless, the patients’ general condition deteriorated, and the patient was suffering from flush symptoms, diarrhea (up to 6x/d) as well as significant weight loss (-3 kg body weight within 4 months). CT-imaging 4 months later revealed further hepatic tumor progression (Fig. 3) along with an elevation in chromogranin A and 5-HIAA concentrations (Fig. 2).

IFNα has been associated to anti-secretory effects in patients with NET, relieving clinical symptoms of carcinoid syndrome. Moreover, it exerts antiproliferative effects in NET providing tumor control in many patients .^[13–15]^ We therefore decided to initiate an add-on therapy with IFN taking into consideration that alternative therapy options such as Peptide receptor radionuclide therapy^[16,17]^ or everolimus^[16]^ were not considered eligible due to negative SSR expression in liver metastases or, in case of everolimus, the lack of anti-secretory effect and minimal cytoreductive ‘potential. Locoablative procedures (Transcatheter arterial chemoembolization, Afterloading, Selective internal radiation therapy) could not be performed based on the high hepatic tumor burden. Due to its better tolerability in comparison to “conventional” IFN, pegylated IFNα2a was administered (135 μg/wk), although it is not specifically approved for NET yet.^[18]^

Following this treatment, the patient fully recovered from signs of hypersecretion and demonstrated an impressive tumor response in CT imaging 4 months later (Fig. 3). Chromogranin A and 5-HIAA concentrations immediately decreased after initiation of therapy as demonstrated in Figure 2. Moreover, the treatment was well tolerated, and the positive effect was not associated with any adverse events. Subsequent staging examinations until December 2019 showed a sustained partial remission and stable peritoneal carcinomatosis. Thus, 30 months after initiation of IFNα-treatment, the patient is in excellent conditions with sustained tumor response and free from any symptoms related to hormone hypersecretion.

## 3 Discussion and conclusion

We report the case of a 63-year-old male patient who was diagnosed in July 2016 with a functional NET of the ileum and synchronous liver metastases. After failure of standard treatment, IFNα therapy was initiated and led to a dramatic clinical response in terms of disappearance of all signs of hormone hypersecretion as well as a sustained tumor shrinkage.

Hypersecretion of neuropeptides is a frequent clinical problem in patients with NET.^[19]^ The so called carcinoid syndrome (CS), mainly consisting of diarrhea, cutaneous flush and respiratory problems was first described in 1954 and later on associated with tumoral secretion of serotonin and histamine.^[20]^ Since CS occurs almost exclusively when liver metastases are present, the treatment-options are palliative in the most cases. According to current guidelines, SSA, interferon-alpha, chemotherapy, loco-regional therapies, target-therapies and peptide recep- tor radionuclide therapy should be considered for treatment of CS.^[21]^ Data from recently published analyses revealed that both lanreotide and octreotide improves diarrhea and flushing in approximately 70% of patients. Notably, lanreotide showed very similar effects as octreotide.^[22,23]^ In patients refractory to a standard SSA first-line treatment, dose escalation or decreasing the injection interval has demonstrated efficacy in many patients and represent the current standard treatment for those patients.^[5,8,11,24]^ Other options for systemic treatment are the use of high-dose SSA^[7,8]^ or of telotristat ethyl, an inhibitor of tryptophan hydroxylase.^[25,26]^ Furthermore, locoregional therapies including palliative surgery, radiofrequency ablation, transarterial embolization and even selective internal radiation therapy have been shown to improve CS.^[5]^ As our patient suffered from progressive hepatic disease and clinical deterioration under intensified SSA therapy and subsequent to palliative resection of the ileal primary tumor, we initiated therapy with IFNα2a.

IFNα has been recommended by many experts and current European guidelines for well differentiated NET as monotherapy or combination therapy with SSA in both antisecretory and antiproliferative intention. Nevertheless, data supporting this recommendation are scarce, the greatest experience is derived from patients with metastatic intestinal NET, with many studies including mixed cohorts of patients with gastroenteropancreatic tumors. In 2 out of 3 randomized trials comparing SSA monotherapy and combination treatment with SSA+ IFN-α, no benefit of the combination therapy could be demonstrated,^[14,27]^ the third study in patients with carcinoid syndrome showed a better tumor control rate in the combination therapy arm, but no significantly better 5-year survival.^[28]^ In a recently published retrospective study, IFN was associated with 15% tumor responses with more than 50% reduction of tumor size, further 39% stable diseases.^[29]^ In our case, thirty months after initiation of IFN treatment our patient still shows complete recovery from clinical symptoms and a sustained biochemical and tumor response going along with a significant reduction in tumor size.

IFNα is associated with considerable toxicity including flu-like symptoms, hematological toxicity, elevated transaminases, nausea, fatigue, and psychiatric sequelae that may significantly limit life quality and may require discontinuation of treatment. Our patient didn’t suffer from any adverse events during the course of treatment. Nevertheless, since the benefit is not proven in large randomized trials and the side-effect profile of other treatments is more favorable than that of IFNα, IFNα should be used only if other treatment options are not available, e.g. in later lines of intestinal NET. In this context, it is important to note that while European guideline recommend use of IFNα in NET as second-line treatment,^[16]^ other international guidelines such as those from North American NET Society (NANETS) do not see an indication for IFNα in these patients due to lack of evidence supporting its use as well as its serious toxic profile.^[14,17]^ In contrast to European guidelines, NANETS guidelines recommend 177Lu-dotatate as convenient regimen for second line treatment ,^[17]^ which was not applicable to our case, as hepatic metastases were negative for SSR.

The exact mechanism driving the antiproliferative effects of IFNα in NET are not clearly understood. Based on data from other tumor entities, it seems likely that the intracellular IFN1 signaling affects cell differentiation, proliferation, and apoptosis. Moreover, recent studies have revealed specific IFN1-regulated genes that may contribute to IFN1-mediated suppression of cancer progression and metastasis.^[30]^ Moreover, it has recently be demonstrated that IFNα activates the cellular autophagy machinery in cell lines and that pharmacological inhibition of autophagy dependent IFNα-induced apoptosis by activation of the CASP8-BID pathway.^[30]^ Finally, modification of the tumor immunology might play a role in mediating effects of IFNα in general as well as in our patient. Considering the dramatic and long-lasting tumor control, it seems plausible that a previously immunologically “cold” tumor was turned “hot” by IFNα and was further controlled by the patients’ immune system. Similar concepts were recently suggested for other substances activating the immune system such as check-point-inhibitors.^[31]^

In summary, this case report supports the use of IFNα in selected patients with functional NET refractory to classical treatments.

## Author contributions

Roderburg and Jann treated the patient and supervised the mansucript.

Özdirik, Tacke and Benz wrote and revised the manuscript.

Amthauer and Fehrenbach provided radiological images and RECIST-Evaluation.
